# Symposium on Vitamin C, 15th September 2017; Part of the Linus Pauling Institute’s 9th International Conference on Diet and Optimum Health

**DOI:** 10.3390/antiox6040094

**Published:** 2017-11-21

**Authors:** Anitra C. Carr

**Affiliations:** Department of Pathology, University of Otago, Christchurch, PO Box 4345, Christchurch 8140, New Zealand; anitra.carr@otago.ac.nz; Tel.: +64-3-364-0649

## 1. Preface

The Linus Pauling Institute’s 9th International Conference on Diet and Optimum Health took place on 13–15 September 2017 in Corvallis, OR, USA, on the beautiful Oregon State University campus. The theme was “Innovative approaches to improving health”, and the event took place on the 100th anniversary of Linus Pauling joining Oregon State University as an undergraduate, and the 20th anniversary of the Linus Pauling Institute’s move to Oregon State from Stanford University. The event also included a tribute to former LPI Director Balz Frei on his retirement (1997–2016). Dr. Frei received the Linus Pauling Institute Prize for Health Research, which is awarded in recognition of innovation and excellence in research relating to the roles of vitamins, essential minerals, and phytochemicals in promoting optimum health and preventing or treating disease, and the roles of oxidative/nitrative stress and antioxidants in human health and disease. The prize also recognizes successful efforts to disseminate and implement knowledge on diet, lifestyle, and health to enhance public health and reduce suffering from disease. I retain fond memories of being the first Postdoctoral Fellow at LPI under Balz Frei’s mentorship (1998–2001). As such, it was my pleasure to convene a special one-day Symposium on Vitamin C (15th September 2017) as a tribute to Balz Frei and the legacy of Linus Pauling. Aside from being awarded two unshared Nobel Prizes, Linus Pauling is perhaps best known for his pioneering work in the 1970s on the role of vitamin C in cancer and infection, so it was fitting that the theme for the symposium was cancer and severe infection (sepsis). Huge breakthroughs have been made in these two fields since Linus Pauling first popularized them. The following abstracts are from the researchers and clinicians who were invited to present at this special symposium on vitamin C, with a focus on mechanisms and therapeutic use in cancer and sepsis.

## 2. Mechanisms of Vitamin C in Cancer

### 2.1. Ascorbic Acid Physiology and Pharmacology: Foundations for Cancer and Sepsis Therapies

LevineMarkMDNational Institute of Diabetes and Digestive and Kidney Diseases, National Institutes of Health, Bethesda, MD 20892, USA; markl@bdg8.niddk.nih.gov

Recommended intakes for many vitamins are based on preventing deficiency, with a safety margin. Our hypotheses are that vitamin recommendations can and should be based on detailed, state-of-the-art physiological investigations in humans, using the clinical tools of concentration-function relationships and pharmacokinetics, coupled with cell biology and genomics. Clinical investigation tools were used to characterize vitamin C physiology in healthy men and women, ages 18–28 years. Utilizing a depletion-repletion design, vitamin C concentrations were found to be tightly controlled in plasma and cells, over a dose range of 30 to 2500 mg. Tight control had at least 4 components: bioavailability, or intestinal absorption; tissue transport; renal filtration, or renal reabsorption/excretion; and utilization. The first three components were regulated by two tissue transporters, mediated by identified transporters SLC23A1 (SVCT1) and SLC23A2 (SVCT2), as well as by as yet unidentified transporters. Recent studies indicate that red blood cells are a unique tissue compartment for ascorbate. In contrast to other tissues, red blood cells transport dehydroascorbic acid (oxidized ascorbate) utilizing the glucose transporter GLUT1. Bioavailability studies showed that percent intestinal absorption decreased as doses increased. Intravenous administration of doses above 100 mg produced ascorbate plasma concentrations that could not be achieved with oral dosing. These data indicated that, depending on dose and rate of administration, intravenous ascorbate produced pharmacologic plasma concentrations, with renal filtration restoring homeostasis. 

Pharmacologic intravenously administered ascorbate, but not oral ascorbate, had the potential to decrease cancer growth in humans. Pharmacologic ascorbate was cytotoxic to cancer, but not to normal cells in vitro, both in animals and in small—but encouraging—studies in humans. Pharmacologic ascorbate mediated cancer cell death by generation of extracellular hydrogen peroxide (H_2_O_2_) in vivo. Pharmacologic ascorbate can be considered a pro-drug for delivery of pharmacologic H_2_O_2_ concentrations to the extracellular space. There is an ever-increasing multiplicity of downstream mechanisms of ascorbate-mediated cancer cell cytotoxicity that are H_2_O_2_-dependent. In specifically modified cell lines, mechanisms have been proposed that are H_2_O_2_-independent; for example, based on dehydroascorbic acid. However, this mechanism does not appear to have general applicability. Clinically, pharmacologic ascorbate has a surprisingly strong safety profile. Non-specificity, or promiscuity, of many oncology therapeutics is often harmful because of collateral damage to normal tissues in humans. In contrast, benefit is provided to patients by the promiscuity of pharmacologic ascorbate because of its safety and potential efficacy. Accelerated ascorbate utilization occurs in critically ill patients. Using similar pharmacokinetics principles as for cancer treatment, intravenous ascorbate has shown recent promise in treatment of sepsis. Based on transporter principles and red blood cell physiology, ascorbate either orally or intravenously has additional promise in delaying complications of diabetic microvascular disease. Considered together, the data indicate that exhaustive characterization of vitamin physiology in healthy people serves as a gateway to advances in disease treatment and prevention. Applying this approach to other vitamins will provide physiologic bases for vitamin recommendations, and may reveal unanticipated application to disease treatment.

### 2.2. Vitamin C-Dependent Regulation of the Hypoxic Response in Cancer

VissersMargreet C.M.PhDDepartment of Pathology, University of Otago, Christchurch 8140, New Zealand; margreet.vissers@otago.ac.nz

Rapid tumor growth initiates hypoxic stress and activates the transcription factor hypoxia-inducible factor (HIF)-1, promoting angiogenesis, glycolysis and enhanced resistance to radio- and chemotherapy. HIF-1 is down-regulated by oxygen-sensing hydroxylases that require vitamin C (ascorbate) as cofactor and we have observed an inverse correlation between cell ascorbate levels and HIF-1 activity. Our hypothesis is that poor vascular ascorbate delivery in a growing tumor limits cell ascorbate content and augments the hypoxic response, thereby driving tumor growth. Raising tumor ascorbate could reverse this effect. Our in vitro modelling of ascorbate uptake into tissues is consistent with this hypothesis and suggests that supra-physiological concentrations are required to saturate tumor tissue. 

We have shown that HIF-1 is moderated by intracellular ascorbate in tumors grown in the Gulo^−/−^ mouse, a model of human vitamin C dependency. Pre-clinical studies with tumor tissue from cancer patients have also shown a correlation between cellular ascorbate levels, HIF-1 activity, tumor size and patient outcome in breast, colorectal, endometrial and renal cancers. These results suggest that raising tumor ascorbate could slow tumor growth by moderating HIF-1 activation. Our data from tumor-bearing Gulo^−/−^ mice suggests that achieving mM plasma ascorbate levels by daily administration of supra-physiological doses exerts an anti-tumor effect, with decreased HIF-1 and vascular endothelial growth factor protein expression as well as reduced microvessel density, tumor hypoxia and tumor growth.

We have recently completed a pilot study with colorectal cancer patients who were given a high dose of vitamin C (1 g/kg) for four days prior to surgical removal of the tumor. Our analysis of the tumor tissue indicates that a significant increase in ascorbate levels is achieved through the high dose intervention that could impact on HIF-1 activation. Together, our results support the suppression of HIF-1 as an anti-tumor activity of ascorbate that may be useful in a clinical setting.

### 2.3. Vitamin C as a Multi-Targeting Anti-Cancer Agent

ChenQiPhDDepartment of Pharmacology, Toxicology and Therapeutics, University of Kansas Medical Center, Kansas City, KS 66160, USA; qchen@kumc.edu

High-dose intravenous ascorbate (IVC) has attracted increasing interests as a low-toxic cancer therapy. IVC bypasses bioavailability barriers of oral ingestion, provides pharmacologic concentrations in tissues, and exhibits selective cytotoxic effects in cancer cells through peroxide formation. The selectivity is related to the mechanisms of action. We postulate that ascorbate-induced reactive oxygen species (ROS) has multiple mechanisms of action that preferably influence cancer cells. First, ascorbate-generated ROS induces DNA damage. Downstream to DNA damage, cellular NAD^+^ decreases as an effect of poly ADP ribose polymerase (PARP) activation. Decrease of NAD^+^ inhibited glyceraldehyde 3-phosphate dehydrogenase (GAPDH) activity and depletes ATP in cancer cells, while normal cells maintain their ATP levels. This phenomenon has its root in dysregulated glucose metabolism in cancer cells, known as the Warburg Effect, whereby cancer cells depend on a larger proportion on glycolysis for ATP, whereas normal cells depend more on oxidative phosphorylation. Second, lack of NAD^+^ inhibits activity of Sirt-2, a tubulin deacetylase, and therefore increases tubulin acetylation, which in turn disrupts dynamics of microtubules. This influences cancer cells that are actively undergoing mitosis and migration. Third, when PARP is inhibited, excessive DNA damage results in cell death. Further, ascorbate inhibited epithelial-mesenchymal transition (EMT), an important process contributing to cancer metastasis. Finally, ascorbate enhanced collagen synthesis in tumor stroma. Despite the controversial reports on the effect of elevated collagen in tumor progression, the increased collagen by ascorbate treatment is associated with restriction of tumor invasion in our animal experiment and in patient.

Taken together, these data show multi-targeting effects of ascorbate that favor death/inhibition in cancer cells relative to normal cells. With minimal toxicity, the multi-targeting mechanism of ascorbate is advantageous because it could decrease the likelihood of resistance, and provides multiple opportunities for combination with standard chemo and radiation therapies. 

### 2.4. Vitamin C and Bone Marrow Stem Cell Transplantation

BosGerard M.J.MDPhDMaastricht University Medical Center, 6202 AZ Maastricht, The Netherlands; gerard.bos@mumc.nl

Bone marrow stem cell transplantation is used in the treatment of patients with cancer, mostly hematological types of cancers. Both autologous transplants and allogeneic transplants can be used. Autologous transplantation is used to recover bone marrow after high-dose chemotherapy, with or without radiotherapy. Allogeneic transplantation is used to induce a graft-versus-tumor response against a patient’s malignant cells, based on an immunological mismatch between donor and recipient. Both treatments do induce a time period of immunosuppression in which the patient is at risk of infections. In autologous transplants, this risk is relatively low (mortality 2–5%). In allogeneic transplants, this risk is higher, and depends partly on the source of the transplant, potentially leading to a state of immune-deficiency of over 3 months.

This clinical question led us to study the possible option of adding back of T and/or Natural Killer (NK) cells cultured from stem cells in vitro. In vitro, it is possible to culture (pre-) T cells from stem cells in the presence of feeder cells transfected with Notch-Ligands, which are crucial for T cell development. In order to test a feeder-free system for GMP-accredited clinical use, we observed that different media gave different results; the difference could be explained by the presence or absence of vitamin C. In the presence of vitamin C, double-positive T cells from stem cells can be obtained in the absence of stromal cells [1]. However, for further development to single positive T cells, stromal cells are still needed. In addition, we also observed that NK cells derived and matured faster from stem cells, as well as from early pre T/NK cells, in the presence of vitamin C [2]. These cells are completely functional NK cells.

Based on these in vitro data, we went back to the clinic to see if patients that are treated for hematological diseases, with or without a stem cell transplantation, might have low vitamin C levels. Patients had lower vitamin C levels (20.4 microMol/L; N = 42) compared to 65 microMol/L in controls [3]. 20% of the patients had levels that were below 11 microMol/L or were undetectable, and were considered to be vitamin C deficient. In a more recent prospective study, we confirm this observation; patients have lower vitamin C levels while on treatment, compared to family members as controls. 

These data are the basis for an intervention study using vitamin C to see if leukocyte recovery, and therefore infections, morbidity and mortality, can be improved after stem-cell transplantation for hematological malignancies.

## 3. Vitamin C Therapy in Cancer

### 3.1. The Chemical Biology of IV-C in Cancer Treatment: Basic Science to Clinical Trials

BuettnerGarry R.PhDDepartment of Radiation Oncology, The University of Iowa, Iowa City, IA 52242-1089, USA; garry-buettner@uiowa.edu

Ascorbate functions as a versatile reducing agent. At pharmacological doses (P-AscH^−^, [plasma] ≈ 20 mM), achievable through intravenous delivery, oxidation of AscH^−^ can produce a high flux of H_2_O_2_ in tumors. Normal cells/tissues seem not to be affected by an increased flux of H_2_O_2_, while exposure to an increased flux of H_2_O_2_ is detrimental to many cancer cells. I will address three basic issues for the use of P-AscH^−^ in the treatment of cancer: (1) the oxidation of ascorbate to produce a flux of H_2_O_2_; (2) catalase as the first-line defense of cells against an increased flux of H_2_O_2_; and (3) potential downstream modulators of the cellular response to this oxidative challenge. Our laboratory is quantitatively addressing these three aspects of the potential use of P-AscH^−^ to treat cancer. I will show how data from experiments that address these issues are used to guide clinical trials. 

The encouraging data from our basic science efforts have underpinned six clinical trials on the use of P-AscH^−^ as an adjuvant to the standard of care at The University of Iowa; one completed (Phase 1, pancreatic cancer with gemcitabine); one terminated (Phase 2, pancreatic cancer with gemcitabine); two active, but recruiting is finished (Phase 1, pancreatic cancer with gemcitabine and radiation; Phase 1, glioblastoma multiforme, temozolomide and radiation); two active and recruiting (Phase 2, Non-Small-Cell Lung Cancer Paclitaxel, Carboplatin); Phase 2, glioblastoma multiforme, temozolomide and radiation). An overview of the current results will be presented.

Conclusions: From our basic science results, P-AscH^−^ may be an effective adjuvant for some standard care therapies; P-AscH^−^ is safe as an adjuvant with the chemotherapeutic agents we have tested, as well as with radiation; adverse events are minimal and mild; there are suggestions of efficacy.

### 3.2. Epigenetic Treatment of Cancer by Vitamin C

WangGaofengPhD*Miller School of Medicine, University of Miami, FL 33136, USA; gwang@med.miami.edu*Unfortunately, Dr. Wang was unable to attend the meeting due to hurricane Irma.

Recent advances have uncovered a previously unknown function of vitamin C in regulating the demethylation of DNA and histones. Ten-eleven translocation (TET) dioxygenases initiate DNA demethylation by converting 5-methylcytosine (5mC) into 5-hydroxymethylcytosine (5hmC). Vitamin C is essential for the function of TET by providing Fe(II), a cofactor of TET. Loss of 5hmC is accompanied by malignant cellular transformation. Overexpressing TET can partially re-establish a normal 5hmC profile in cancer cells and represses their malignancy. While overexpressing TETs in patients might not be clinically feasible, these discoveries suggest that finding a means of restoring normal 5hmC content may yield a novel therapy for cancer. The expression of vitamin C transporter SVCT2 is frequently downregulated in cancer. For instance, SVCT2 expression is decreased in 72.5% of breast cancer cases by at least 1.5-fold compared to the matched normal breast tissues. This suggests that it is necessary to compensate the downregulated SVCT2 with vitamin C supplements in at-risk populations and cancer patients. Treatment of cancer cells with vitamin C increases 5hmC content and results in a markedly shifted transcriptome. These changes are correlated with decreased cellular malignant phenotypes. Furthermore, by promoting the demethylation of DNA and histones, vitamin C changes the response of cancer treatment. For example, vitamin C improves the efficacy of Bromodomain and extraterminal domain inhibitors (BETi) in treating melanoma. In conclusion, vitamin C can prevent cancer initiation and progression by reestablishing 5hmC. Vitamin C also can improve the response of certain cancer drugs.

### 3.3. IVC and Chemotherapy in Ovarian Cancer

DriskoJeanneMDUniversity of Kansas Medical Center, Kansas City, KS 66160, USA; jdrisko@kumc.edu

Background: Ascorbate (vitamin C) has long been used as an unorthodox therapy for cancer, even though the underlying scientific mechanisms are not well understood.

Clinical Trial: A pilot phase 1/2a clinical trial was conducted in patients with newly diagnosed stage III or IV ovarian cancer. High-dose intravenous ascorbate (AA) was added to conventional paclitaxel/carboplatin (Pax + Cp) therapy, and toxicity was assessed. Twenty-seven participants were randomized into either the standard Cp + Pax arm or the Cp + Pax + AA arm. Cp + Pax chemotherapy was administered for the initial 6 months, and AA treatment for 12 months. Any and all unwanted events were counted and graded for severity according to NCI CTCAEv3. Records for adverse events include patient interviews, emergency room visits, patients’ oncologist visits, and hospitalization records. The number of adverse events in each grade for each participant was divided by the number of encounters of that participant, and then the adverse events per encounter were averaged in the Cp + Pax arm and the Cp + Pax + AA arm, respectively. Participants were followed for survival for 5 years.

Statistical Analysis: Two-tailed Student’s *t* test was performed for toxicity comparison between chemotherapy group and chemotherapy + ascorbate group. Welch’s *t* test was used when the variances in the two compared populations were unequal. A log-rank test was performed for comparison of the survival curves between the chemotherapy group and the chemotherapy + ascorbate group.

Results: Ascorbate worked synergistically in vitro and in vivo with the first-line chemotherapeutic drugs carboplatin and paclitaxel. In patients with advanced ovarian cancer, treatment with ascorbate reduced toxicities associated with chemotherapy. Because the study was not powered for detection of efficacy, statistical improvement in survival was not observed.

Conclusion: Given the advantage of low toxicity of ascorbate, larger clinical trials need to be done to definitively examine the benefit of adding ascorbate to conventional chemotherapy.

### 3.4. Challenges of Natural Product Drug Development

PallerChanningMDDepartment of Oncology, Johns Hopkins University School of Medicine, Baltimore, MD 21205, USA; cpaller1@jhmi.edu

Numerous drugs that the US Food and Drug Administration (FDA) has approved for use in cancer therapy are derived from plants, including taxanes such as paclitaxel and vinca alkaloids such as vinblastine. Dietary supplements are another category of natural products that are widely used by patients with cancer, but without the FDA-reviewed evidence of safety and efficacy—be it related to survival, palliation, symptom mitigation, and/or immune system enhancement—that is required for therapy approval. Nearly half of patients in the United States with cancer report that they started taking new dietary supplements after being given a diagnosis of cancer. Oncologists are challenged in providing advice to patients about which supplements are safe and effective to use to treat cancer or the side effects of cancer therapy, and which supplements are antagonistic to standard treatment with chemotherapy, radiation, and/or immunotherapy. Despite the large number of trials that have been launched, the FDA has not approved any dietary supplements or food for the prevention of cancer, to halt its growth, or to prevent its recurrence. We will review the primary challenges faced by researchers attempting to conduct rigorous trials of natural products, including shortages of funding due to lack of patentability, manufacturing difficulties, contamination, and lack of product consistency. We will also highlight the methods used by dietary supplement marketers to persuade patients that a supplement is effective (or at least safe) even without FDA approval, as well as the efforts of the US government to protect the health and safety of its citizens by ensuring that the information used to market natural products is accurate. Finally we will close with a summary of the most widely used databases of information about the safety, efficacy, and interactions of dietary supplements.

## 4. Vitamin C Therapy in Sepsis

### 4.1. IVC in Pre-Clinical Sepsis and Trauma Models

NatarajanRameshPhDDepartment of Internal Medicine, Virginia Commonwealth University School of Medicine, Richmond, VA 23298-0663, USA; ramesh.natarajan@vcuhealth.org

Bacterial infections of the lungs and abdomen are among the most common causes of sepsis. Sepsis-induced acute lung injury (ALI) is a persisting clinical problem with no direct therapy. We used various models of sepsis in wild-type and knockout mice to determine whether parenteral vitamin C modulates the dysregulated pro-inflammatory, pro-coagulant state that leads to sepsis-induced lung injury. Male C57BL/6 wild type mice and mice lacking functional l-gulono-γ-lactone oxidase (Gulo^−/−^) were exposed to bacterial lipopolysaccharide (endotoxin) or a fecal stem solution (polymicrobial sepsis) to induce abdominal peritonitis 30 min prior to parenterally receiving either reduced vitamin C (ascorbic acid, 200 mg/kg) or oxidized vitamin C (dehydroascorbic acid, 200 mg/kg). Outcomes examined included survival, extent of ALI, pulmonary inflammatory markers, bronchoalveolar epithelial permeability, alveolar fluid clearance, epithelial ion channel, and pump expression, tight junction protein expression, cytoskeletal rearrangements, neutrophil extracellular trap formation (NETosis), multiple organ failure and various coagulation parameters in whole blood. Sepsis-induced ALI was characterized by compromised lung epithelial permeability, reduced alveolar fluid clearance, pulmonary inflammation and neutrophil sequestration, increased formation of NETs, significant viscoelastic changes in blood, and increased mortality due to multiple organ failure. A single infusion of parenteral vitamin C protected mice from the deleterious consequences of sepsis by multiple mechanisms, including attenuation of the NFκB driven pro-inflammatory response, enhancement of epithelial barrier function, increasing alveolar fluid clearance, restoration of endothelial function, prevention of sepsis-associated coagulation abnormalities, attenuation of NETosis, and normalization of physiological functions that attenuated the development of multiple organ dysfunction. These pre-clinical studies using parenteral vitamin C were central to a completed PHASE I trial of intravenous vitamin C in sepsis patients and an ongoing PHASE II multi-center trial examining the efficacy of intravenous vitamin C infusion in human sepsis-associated ALI.

Proteomic analysis of plasma from a model of hemorrhagic shock and polytrauma in swine showed that IV vitamin C may mitigate the pro-inflammatory/pro-coagulant response that contributes to multiple organ failure following acute severe polytrauma by maintaining circulating levels of ADAMTS13. ADAMTS13 is a disintegrin-like metalloprotease that cleaves and inactivates von Willebrand Factor, a pro-coagulant molecule that is released from activated endothelial cells and promotes platelet activation.

### 4.2. Intravenous Vitamin C as Therapy for Sepsis-Induced Acute Lung Injury

(Berry) FowlerAlpha A.IIIMDDepartment of Internal Medicine, Virginia Commonwealth University School of Medicine, Richmond, VA 23298-0663, USA; alpha.fowler@vcuhealth.org

The incidence of sepsis and sepsis-associated organ failure continues to rise in American Intensive Care Units. Over 1 million cases of sepsis (i.e., bacterial, fungal, viral) occur in the U.S. population each year. Some 375,000 patients will die from sepsis, either primarily from septic shock or secondarily from organ failure. Sepsis induced organ failure contributes cumulatively to patient mortality. Patients with severe sepsis suffer higher mortality rates compared to patients with organ failure but no sepsis. Despite over 15,000 patients studied, and over 1 billion dollars in study costs, effective sepsis therapy remains elusive. Clinical trials that have targeted mediators of inflammation or coagulation such as rosuvastatin or activated protein C have not reduced septic mortality, suggesting that single-target therapy fails to meet the challenges of complex multicellular activation and interactions. Recent studies suggest that ascorbic acid may attenuate pathological responses in septic microvasculature. In preclinical studies, ascorbic acid improved capillary blood flow, microvascular barrier function, and arteriolar responsiveness to vasoconstrictors in septic animals. We showed that parenterally administered ascorbic acid attenuated vascular lung injury in septic mice. Subnormal plasma ascorbic acid concentrations in septic patients correlates inversely with multiple organ failure and directly with survival. We report, in this presentation, that intravenous vitamin C is safe to administer to patients with severe sepsis, that it improves sepsis-induced organ failure, and that it attenuates biomarkers of systemic inflammation and vascular injury. We also report aspects of the ongoing NIH-sponsored trial: Vitamin C Infusion for TReatment In Sepsis Induced Acute Lung Injury (CITRIS-ALI).

### 4.3. Vitamin C Requirements and Mechanisms of Action in Severe Infection

CarrAnitra C.PhDDepartment of Pathology, University of Otago, Christchurch 8140, New Zealand; anitra.carr@otago.ac.nz

Patients with severe infections such as pneumonia can develop sepsis, an uncontrolled inflammatory response to the initial infection. This can result in organ failure and septic shock, the major cause of death of critically ill patients in intensive care. We and others have found that critically ill patients have severely depleted vitamin C levels, despite recommended intakes via liquid nutrition [4]. One study has shown that critically ill patients require parenteral vitamin C at levels that are 30-fold higher than recommended intakes. Patients with sepsis have dysregulated immune function, including compromised leukocyte function. Neutrophils are the primary responders to infection, and these cells are known to accumulate high levels of vitamin C, suggesting an important role for the vitamin in immune cell function. We and others have shown that vitamin C can enhance neutrophil chemotaxis, oxidant production, apoptosis and clearance by macrophages [5]. It is possible that the dramatic clearance of septic lungs observed following vitamin C treatment could be partly due to enhanced clearance of apoptotic neutrophils. Vitamin C is an important cofactor for numerous biosynthetic and regulatory enzymes in the body. Because vitamin C is a cofactor for the enzymes that synthesize noradrenaline and vasopressin, we hypothesized that vitamin C administration to patients with severe sepsis and septic shock may decrease the need for exogenous administration of these vasopressors [6]. Support for the vitamin C and vasopressor hypothesis comes from recent clinical trials that showed decreased vasopressor requirements in patients who received intravenous vitamin C. We are currently implementing a clinical trial in Christchurch Hospital ICU to assess the outcomes and mechanisms of action of intravenous vitamin C in severe sepsis.

### 4.4. Vitamin C, Hydrocortisone and Thiamine for the Treatment of Severe Sepsis and Septic Shock: The Metabolic Resuscitation Protocol

MarikPaul E.PhDDepartment of Internal Medicine, Eastern Virginia Medical School, Norfolk, VA 23501-1980, USA; marikpe@evms.edu

A large body of experimental data has demonstrated that both corticosteroids and intravenous vitamin C reduce activation of nuclear factor κB (NF-κB), attenuating the release of pro-inflammatory mediators; reduce the endothelial injury characteristic of sepsis, thereby reducing endothelial permeability and improving microcirculatory flow; augment the release of endogenous catecholamines; and enhance vasopressor responsiveness. In animal models, these effects have resulted in reduced organ injury and increased survival. Corticosteroids have been evaluated in several clinical trials, with meta-analysis of these trials demonstrating somewhat conflicting outcomes. Low-dose stress corticosteroids have proven to be safe, with no increased risk of clinically important complications. While corticosteroids decrease vasopressor dependency, the effect on the risk of developing organ failure and survival is less clear. Similarly, intravenous vitamin C has been evaluated in unselected surgical ICU patients, patients with burns, those with pancreatitis, and in two pilot studies of patients with severe sepsis and septic shock [7]. In general, these studies have demonstrated a reduction in the risk of multisystem organ failure (MSOF), although the effect on mortality is less clear. However, IV vitamin C has been shown to be extremely safe, with no recorded complications.

In vitro data has suggested that vitamin C and hydrocortisone may act synergistically. Barabutis et al. have demonstrated that hydrocortisone, together with vitamin C, protects the vascular endothelium from damage by endotoxin while neither agent alone had this effect [8]. Based on these clinical and experimental data, we initiated a treatment protocol for patients with severe sepsis and septic shock that included intravenous vitamin C, hydrocortisone and thiamine. We have demonstrated that this therapeutic cocktail reverses the organ dysfunction of sepsis with a marked reduction in mortality [9].
